# Reply to “Early resolution of subretinal fluid without high-dose corticosteroids in a pregnant patient with Vogt-Koyanagi Harada disease” by Sugita et al.

**DOI:** 10.1186/s12348-016-0080-5

**Published:** 2016-05-03

**Authors:** Kalpana Babu, Aditi Parikh

**Affiliations:** Vittala International Institute of Ophthalmology & Prabha Eye Clinic and Research Center, 504, 40th Cross, Jayanagar 8th block, Bangalore, 560070 India

**Keywords:** Resolution, Vogt-Koyanagi-Harada disease, Pregnancy, Corticosteroids

## Abstract

We read with great interest the article by Sugita et al. on early resolution of subretinal fluid without high-dose corticosteroids in a pregnant patient with Vogt-Koyanagi-Harada disease. We would like to share a similar experience where the subretinal fluid resolved within 2 weeks without treatment in a pregnant woman who was in her second trimester.

Dear editor,

We read with great interest the brief report by Sugita et al. [1] regarding “Early resolution of subretinal fluid without high-dose corticosteroids in a pregnant patient with Vogt-Koyanagi-Harada disease.” We would like to share a similar case who presented to our clinic recently. A 31-year-old woman in the second trimester of pregnancy (24 weeks) presented with sudden blurring of vision in both eyes of 2 days duration. She had a vague history of headache. Her BCVA was 20/120 and 20/200 in OD and OS, respectively. Slitlamp examination was normal. Fundus examination showed occasional vitreous cells and bilateral, multifocal serous retinal detachments (Fig. 1a, b). Intraocular pressures were normal. OCT confirmed the foveal neurosensory detachments (Fig. 2a, b). Choroidal thickness was 1.6 and 1.8 mm in OD and OS, respectively. Fluorescein angiography was not done in view of the pregnancy. Her BP was 120/70 mm of Hg. Routine laboratory investigations to rule out other causes of uveitis did not reveal any abnormality. She had no history of any systemic disease. Based on the clinical findings, she was diagnosed to have incomplete VKH and was recommended systemic corticosteroids. However, the patient refused treatment because she was worried about adverse effects to the fetus. She came to us 2 weeks later with a dramatic improvement in her symptoms and ocular signs. Her BCVA was now 20/30 (OU). Early depigmentary alterations were now seen in the retinal periphery (Fig. 3a–c). She was lost to follow-up thereafter. We learnt from her treating obstetrician, she had delivered a healthy male baby 3 months later. What is interesting is the occurrence of inflammation in the second trimester of pregnancy and the rapid resolution of fluid within 2 weeks without treatment, in a patient without a prior history of VKH. However, unlike the case by Sugita et al. [1], our patient did not have any systemic disease and did not receive any corticosteroids in the past. Though it is difficult to explain the observation seen, this case in addition to the case reported by Sugita et al. [1] has shown early resolution of subretinal fluid without treatment in VKH leading us to revisit the role of pregnancy in VKH.

**Fig. 1:**
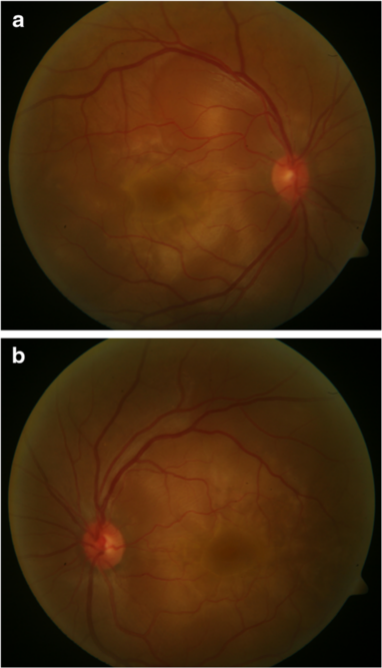
Fundus photograph of the right (**a**) and left (**b**) eyes showing multiple serous retinal detachments

**Fig. 2:**
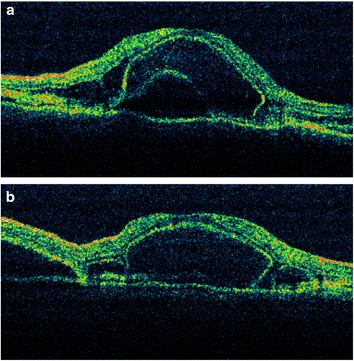
Optical coherence tomography of the right (**a**) and left (**b**) eyes showing the serous retinal detachments

**Fig. 3:**
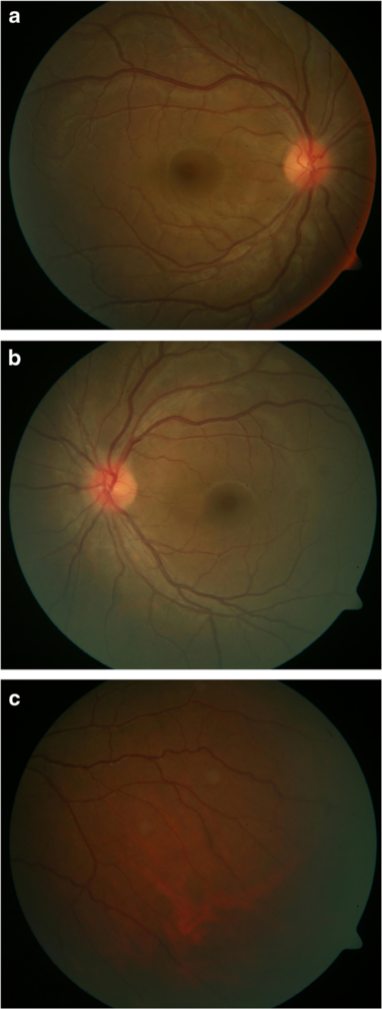
Fundus photograph of the right (**a**) and left (**b**) eyes showing resolution of subretinal fluid and early depigmentary alterations (**c**)

## Abbreviations

BCVA: best corrected visual acuity
BP: blood pressure
OD: right
OS: left
OU: both
VKH: Vogt-Koyanagi Harada disease
